# webPRANK: a phylogeny-aware multiple sequence aligner with interactive alignment browser

**DOI:** 10.1186/1471-2105-11-579

**Published:** 2010-11-26

**Authors:** Ari Löytynoja, Nick Goldman

**Affiliations:** 1 EMBL-European Bioinformatics Institute, Hinxton, Cambridgeshire, UK

## Abstract

**Background:**

Phylogeny-aware progressive alignment has been found to perform well in phylogenetic alignment benchmarks and to produce superior alignments for the inference of selection on codon sequences. Its implementation in the PRANK alignment program package also allows modelling of complex evolutionary processes and inference of posterior probabilities for sequence sites evolving under each distinct scenario, either simultaneously with the alignment of sequences or as a post-processing step for an existing alignment. This has led to software with many advanced features, and users may find it difficult to generate optimal alignments, visualise the full information in their alignment results, or post-process these results, e.g. by objectively selecting subsets of alignment sites.

**Results:**

We have created a web server called webPRANK that provides an easy-to-use interface to the PRANK phylogeny-aware alignment algorithm. The webPRANK server supports the alignment of DNA, protein and codon sequences as well as protein-translated alignment of cDNAs, and includes built-in structure models for the alignment of genomic sequences. The resulting alignments can be exported in various formats widely used in evolutionary sequence analyses. The webPRANK server also includes a powerful web-based alignment browser for the visualisation and post-processing of the results in the context of a cladogram relating the sequences, allowing (e.g.) removal of alignment columns with low posterior reliability. In addition to *de novo *alignments, webPRANK can be used for the inference of ancestral sequences with phylogenetically realistic gap patterns, and for the annotation and post-processing of existing alignments. The webPRANK server is freely available on the web at http://tinyurl.com/webprank .

**Conclusions:**

The webPRANK server incorporates phylogeny-aware multiple sequence alignment, visualisation and post-processing in an easy-to-use web interface. It widens the user base of phylogeny-aware multiple sequence alignment and allows the performance of all alignment-related activity for small sequence analysis projects using only a standard web browser.

## Background

When used for evolutionary or phylogenetic analyses, a sequence alignment is meant to represent evolutionary homology and have the characters descended from a common ancestor placed in the same column. Commonly used alignment programs do not consider phylogeny in their placement of gaps, however, and create systematic errors with insertion-deletion events, affecting downstream evolutionary analyses [1,2]. The phylogeny-aware algorithm that distinguishes insertions from deletions [3] has been shown not to suffer from this bias and, when the input guide phylogeny can be trusted, produces superior inference of character homology [2]. The alignments generated using the algorithm have been found to perform well in phylogenetic alignment benchmarks based on both real [4] and simulated [2] data, and give the most accurate inference of selection on codon sequences [5].

**Figure 1:**
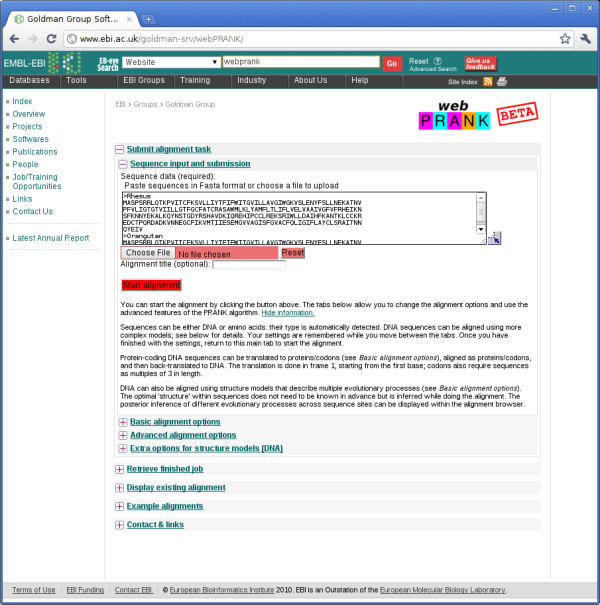
**The webPRANK submission page has a modular, easy-to-use interface and contains integrated documentation explaining the main features**. In addition to submission of new tasks and retrieval of finished jobs, the site allows uploading of existing alignments to a web-based alignment browser for visualisation and post-processing.

**Figure 2:**
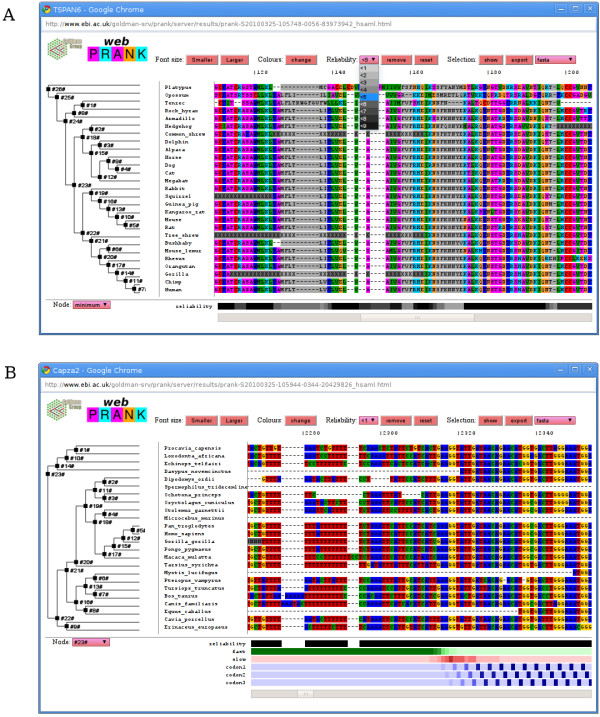
**Resulting alignments, and uploaded alignments in HSAML format, can be displayed and post-processed using a powerful alignment browser**. The alignments are shown alongside the guide tree relating the sequences; the tree is interactive and allows control of the information tracks displayed. **(A) **The site-wise alignment reliability is indicated in shades of grey (bottom). Unreliably aligned sites are deselected (columns marked grey) using an adjustable threshold (drop-down 'Reliability' menu) and the remaining sites can be exported in various alignment formats for downstream evolutionary analyses. **(B) **Using the FAST/SLOW/CODON model, inferred sequence structure for genomic DNA alignments is indicated in shades of green, red and blue (bottom). A prediction for a pre-computed EPO alignment [16] shows a correctly inferred change from the FAST state (green) in the intron through the SLOW state (red) at the splice site to the three CODON states (blue) in the exon.

The phylogeny-aware algorithm, including support for structure models describing differently-evolving site classes [6], has been implemented in the PRANK alignment software and its graphical interface PRANKSTER (both freely available from http://tinyurl.com/prank-msa). We now add an easy-to-use web interface called webPRANK (Figure 1), and provide a powerful web-based browser to visualise and post-process the resulting alignments (Figure 2). The new interface, with integrated documentation, can be accessed at http://tinyurl.com/webprank .We hope webPRANK will encourage a wide range of researchers to consider explicitly the evolution of the sequences they align and to properly account for gaps.

## Implementation

The webPRANK server is based on the PRANK phylogeny-aware multiple sequence alignment software [3], implemented in C++. The alignment tasks are executed at the European Bioinformatics Institute's computation cluster using Web Services [7], supporting delayed retrieval of finished jobs. The webPRANK user interface is written in HTML and JavaScript and the server back-end with Perl, using CGI and the SOAP messaging protocol for communication and accessing the web service.

The webPRANK server uses the XML-based HSAML sequence alignment format (defined in http://tinyurl.com/hsaml) extensively. The format is capable of storing the full information of the alignment process, including the guide tree and the posterior scores associated with the pair-wise alignments at its internal nodes, in a single, computer-parsable file. This enables advanced analysis and post-processing of newly-generated results as well as full re-processing of earlier results, stored locally in the format, within the associated alignment browser.

The webPRANK alignment browser provides a powerful interface to analyse and post-process the results within a standard web browser. Its interactive functions are implemented using advanced features of JavaScript and their full functionality requires a web browser with a fast JavaScript engine. The processing of large data sets can become computationally heavy and an alternative browser is provided for basic data visualisation. This light-weight alignment viewer, implemented in plain HTML, allows simple analyses with a web browser not supporting the full JavaScript or using a less powerful computer.

## Results

The webPRANK server (Figure 1) supports the alignment of DNA, protein and codon sequences, input in FASTA format [8], using evolutionary substitution models [9-11]. It can translate, align as protein and back-translate protein-coding DNA sequences. In addition, webPRANK includes built-in support for two structure models [6], FAST/SLOW and FAST/SLOW/CODON, designed for aligning genomic DNA sequences with sites evolving with different substitution dynamics and differences in the patterns of alignment gaps. webPRANK accepts a user-defined phylogeny (Newick format) to guide its progressive alignment procedure, or can compute one from the unaligned input sequences. For each alignment task, the full combination of parameters, and the structure model if used, are provided in the output so that the analyses can easily be repeated or recreated with the stand-alone PRANK program.

The size of alignment tasks is limited to 4 GB of memory and 24 hours of run time. The size and type of data as well as the parameter settings affect the computation time. The PRANK algorithm has time complexity *O*(*a*^2^*nl*^2^) where *a*, *n *and *l *are the size of the character alphabet (four for DNA; 20 for amino acids; 61 for codons) and the number and length of sequences, respectively. (More precisely, *l *is the length of the sub-alignments to be aligned and, for large *n*, can be much longer than any of the extant or inferred ancestral sequences.) The alignment of 30 DNA sequences of ~1000 nucleotides typically takes 1-2.5 minutes depending on the options chosen; that of 100 DNA sequences of similar length 3.5-20 minutes. The translation of DNA sequences to amino acids or codons decreases sequence lengths but increases alphabet size, requiring computation times similar to (for amino acids) or significantly longer than (for codons) those for untranslated DNA sequences. By default, webPRANK uses alignment anchoring to accelerate analyses of long DNA sequences.

Significant proportions of the longer time estimates for the alignment tasks are spent computing the guide trees and, if a user-defined phylogeny is provided, even larger data sets can be aligned in a reasonable time. With a pre-defined guide tree, the alignment of 1000 simulated DNA sequences of ~1000 nucleotides could be performed in 35 minutes; however, the alignment matrix was 7247 columns wide (the correct width was 7235 columns) and so sparse that it was largely unreadable (see Additional file 1). In practice webPRANK is able to align and display (see below) almost any set of sequences for which subsequent alignment browsing is feasible, and many realistic sets for which it is not.

The webPRANK-generated alignments can be downloaded in several alignment formats widely used in evolutionary analyses. The webPRANK server supports its own HSAML format, as well as FASTA [8], PHYLIP (interleaved and sequential) [12], PAML [13] and NEXUS [14] formats. The XML-based HSAML format is the only one we know that can contain the full information of the alignment process and allows for advanced analysis and post-processing of the results with the integrated webPRANK browser or using the stand-alone PRANKSTER alignment browser. The format can also be easily parsed using external software, for example the XML library for the R statistics package [15] or the libXML module for the Perl programming language, allowing for complex downstream analyses of the alignment data. Of the classical alignment formats, the NEXUS format also allows incorporation of some additional information in the alignment files: webPRANK extends alignments exported in NEXUS format to include the alignment guide tree and the column-wise minimum posterior reliability scores or the excluded alignment sites (see below) using the appropriate commands in the 'Trees', 'Assumptions' and 'Paup' blocks, respectively.

Before downloading the results, the sequence alignments can be visualised and post-processed using a powerful, integrated alignment browser (Figure 2). A distinctive feature of the webPRANK browser is the display of an interactive cladogram, representing the alignment guide tree, next to the sequences. The tree has two purposes. First, we believe that evolutionary sequence alignment should always be studied in the context of the tree relating the sequences. The fact that the guide tree used for the alignment may not be fully correct does not change this, as the tree has nevertheless been used for the alignment and the solution depends on it. Rather than hiding the tree, showing it alongside the alignment helps to identify possible errors and suggest actions to correct them. Second, the PRANK alignments contain additional information associated with the tree nodes and the easiest way to represent and allow browsing this information is in the context of the tree.

The interactive webPRANK browser uses advanced features of JavaScript and requires a modern web browser such as Firefox version 3+, Safari 4+ or Chrome to work properly. The processing of sequences can be computationally heavy, however, and the browser may open with a small delay. The delay is not significant for small alignments and should be bearable for medium-sized alignments (up to 100-200 sequences and a few thousand columns). For larger alignments (up to several thousands of sequences), the webPRANK server provides an alternative, light-weight browser that allows basic horizontal and vertical scrolling of the alignment matrices. Alternatively, the full results can be downloaded in HSAML format and analysed locally using the stand-alone PRANKSTER program, or the aligned sequences exported in simpler formats for visualisation with other browser software.

The PRANK algorithm can compute column-wise reliability scores for the alignment and, when a structure model is used, provide posterior probabilities for the alignment sites evolving under different evolutionary processes [6]. The reliability and probability values are generated by the pair-wise alignments at the different levels of the progressive alignment and are thus associated with the internal nodes of the tree. The information is displayed below the alignment as probability tracks (Figure 2). The tracks for different stages of the alignment can be selected by clicking the corresponding nodes in the tree or using the drop-down menu.

The PRANK alignment reliability scores provide an objective measure to remove less reliably aligned columns from the data and the webPRANK browser includes advanced functionality to select sets of alignment sites using these scores. The webPRANK filtering is based on the track currently displayed; repeated steps of filtering are accepted and, for convenience, an additional track showing the minimum reliability score across all pair-wise alignments is provided. The current selection of alignment sites is indicated in the browser window using different colouring (Figure 2A) and the subset of sites currently selected can be exported in various different alignment formats for the downstream analyses. Unlike other export formats that permanently remove unreliable columns from the data, the files saved in NEXUS format keep the full alignment data and include additional commands excluding a set of sites in the downstream analysis.

As a part of the alignment process, the PRANK algorithm reconstructs the sequence history with inferred ancestral nodes. The inferred ancestral sequences, with phylogenetically realistic patterns of character presence vs. absence, can be displayed in the alignment browser or downloaded for further analyses. Ancestral sequences can also be inferred from existing alignments. One should note, however, that non-phylogeny-aware alignment algorithms tend to infer excess deletions [2] and inference from systematically incorrect alignments typically produces unrealistically long ancestral sequences. In addition to ancestral sequences, structure predictions and alignment reliability scores can also be computed for existing alignments (Figure 2B). This allows application of some of the advanced features of the PRANK alignment package to other alignments, e.g. for objectively removing noise from the alignment data.

The webPRANK alignment browser is not limited to the display of *de novo *alignments: it can be used for visualisation and browsing of any FASTA- or HSAML-formatted alignment, although the full functionality of the browser requires the richer HSAML format. By storing webPRANK-generated alignments in this format, the user can later re-load the results to the webPRANK browser for visualisation and post-processing, and thus perform all alignment-related activity for small sequence analysis projects using a standard web browser only.

## Conclusions

The webPRANK server incorporates phylogeny-aware multiple sequence alignment, visualisation and post-processing. It widens the user base of phylogeny-aware multiple sequence alignment, which can lead to superior inference of character homology and downstream evolutionary analyses. We encourage the consideration of evolutionary sequence alignment in the context of the tree relating the sequences, and the use of enhanced alignment formats such as HSAML for the exchange of linked phylogenetic and alignment information. Our various alignment-related software and format definitions are all available via http://www.ebi.ac.uk/goldman-srv/prank as well as at the URLs listed above.

## Availability and Requirements

Project name: webPRANK

Project home page: http://www.ebi.ac.uk/goldman-srv/webPRANK/

Operating systems: Platform independent (web server)

Programming language: C++ (PRANK alignment software), Perl/CGI/SOAP (server interface),

HTML/JavaScript (web site, alignment browser)

Licence: GNU GPL (PRANK alignment software)

Any restrictions to use by non-academics: none

## Authors' contributions

NG initiated the project and participated in its design, testing and coordination. AL implemented the ideas, wrote the software and drafted the manuscript. Both authors reviewed and approved the final manuscript.

## Supplementary Material

Additional file 1 **The document contains a figure illustrating the difficulty of browsing alignments of many sequences, even when they are closely related, and text explaining this figure**. Click here for file
