# Supplementary material for: webPRANK: a phylogeny-aware multiple sequence aligner with interactive alignment browser
Source: BMC Bioinformatics. 2010 Nov 26;11:579. doi: 10.1186/1471-2105-11-579 (PMC3009689; doi:10.1186/1471-2105-11-579)
Supplement: Additional file 1 — The document contains a figure illustrating the difficulty of browsing alignments of many sequences, even when they are closely related, and text explaining this figure. [file 1471-2105-11-579-S1.PDF]

webPRANK: a phylogeny-aware multiple sequence aligner with interactive alignment browser

Ari Löytynoja*and Nick Goldman

EMBL-European Bioinformatics Institute, Hinxton, Cambridgeshire, UK

Email: Ari Löytynoja*- ari@ebi.ac.uk; Nick Goldman - goldman@ebi.ac.uk;

*Corresponding author

Additional material: browsing alignments of many sequences

(A) We simulated an ancestral DNA sequence of 1000 nucleotides and its evolution over the phylogeny shown (left) to derive 1000 related sequences each of length ∼1000 nucleotides (clock-like tree; root to tip distance = 0.1 expected nucleotide substitutions per site; insertion and deletion rates 0.08 and 0.12 times the substitution rate, respectively; insertion and deletion lengths following a geometric distribution with mean 1.667), using the INDELible program (Fletcher and Yang, 2009, *Mol. Biol. Evol.* 26: 1879–1888). These represent a large number of closely related sequences with moderate levels of insertion and deletion, which are not difficult to align accurately with a phylogeny-aware aligner such as webPRANK. However, despite the relatively low pairwise distance between any two sequences, the large number of sequences makes the total tree length very large, 46.91 substitutions per site, and we expect to see, in average, 3.75 independent insertions between every two consecutive characters of the ancestral sequence. The true alignment (right) appears very sparse; it contains 7235 columns, even though each individual sequence has ∼1000 nucleotides (mean 993.9; range 966–1032), and is not amenable to useful viewing with webPRANK's integrated browser or any other browser we know. This is because of the ∼3740 unrelated insertions that have occurred. Nevertheless, there is a large amount of evolutionary information in this alignment. In **(B)** we show the 'sub-alignments' resulting from choosing a clade of five closely related sequences (top), adding to this another similar clade such that the common ancestor is near the root of the entire 1000-sequence tree (middle), and finally combining five such clades (bottom). No re-alignment has taken place; we have simply extracted the relevant rows from the full alignment in (A) and discarded columns containing only gap characters. The sub-alignments are shown to the same scale as in (A) (their actual sizes are 1002, 1032 and 1099 columns, respectively) to emphasise the generation of 'sparseness' by the inclusion of very large numbers of related sequences, even closely related ones each with low levels of insertion. These sub-alignments are redrawn to a larger scale in **(C)**. This permits their phylogenies to be displayed (left) and shows that there is indeed a very high level of evolutionary information across even the most divergent sequences of the 1000-sequence alignment, a fact that is masked if only view (A) is considered.

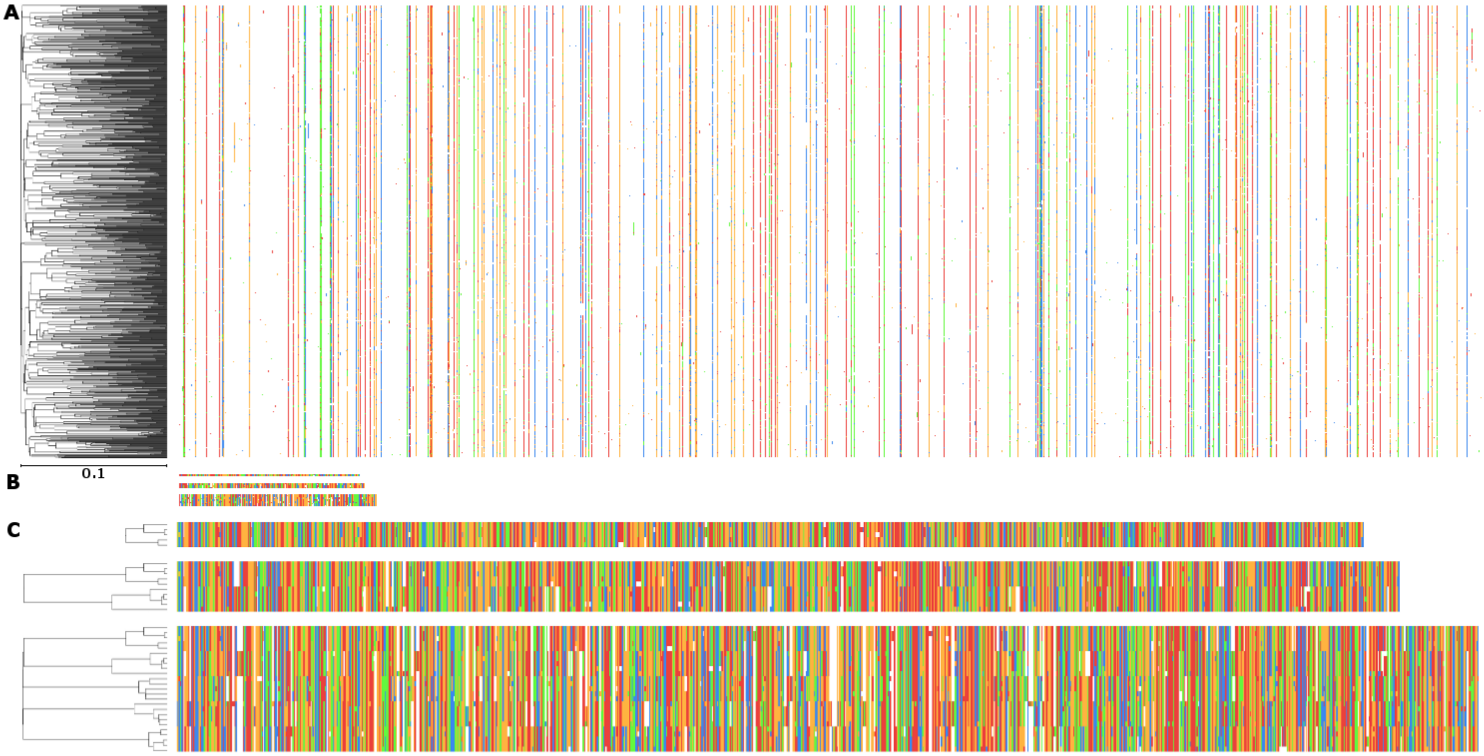
A
0.1
B
C
